# Strain Rate-Dependent Hyperbolic Constitutive Model for Tensile Behavior of PE100 Pipe Material

**DOI:** 10.3390/polym14071357

**Published:** 2022-03-27

**Authors:** Yan Li, Wenbo Luo, Maodong Li, Bo Yang, Xiu Liu

**Affiliations:** 1 College of Civil Engineering and Mechanics, Xiangtan University, Xiangtan 411105, China; liyanningmeng@163.com; 2 Department of Civil Engineering, Changsha University, Changsha 410022, China; 3 Hunan Key Laboratory of Geomechanics and Engineering Safety, Xiangtan University, Xiangtan 411105, China; 4 Guangzhou Special Pressure Equipment Inspection and Research Institute, Guangzhou 510663, China; lmd92791@21cn.com (M.L.); boycs@126.com (B.Y.)

**Keywords:** high-density polyethylene, rate dependence, hyperbolic constitutive model, yield stress, yield strain, initial tangent modulus

## Abstract

It is not conservative to directly use the strength tested under the laboratory loading rates to evaluate the long-term creep strength of polymers. A suitable strain rate-dependent constitutive model is crucial for accurately predicting the long-term strength and mechanical behavior of polymer pressure pipes. In this study, the Kondner hyperbolic constitutive model is considered the base model in deriving the rate-dependent constitutive model for PE100 pipe material, and the yield stress and initial tangent modulus are the two rate-dependent parameters of the model. Uniaxial tension tests are carried out under five specified strain rates ranging from 10^−5^ s^−1^ to 5 × 10^−2^ s^−1^ to obtain these two parameters. It is demonstrated that the strain rate dependence of the yield stress and the initial tangent modulus can be described by either a power or a logarithm law. The predictions from the two models are in good agreement with the experiments. In contrast, the power-law rate-dependent Kondner model is more suitable for describing the rate-dependent tensile behavior of PE100 pipe material than the logarithm-law rate-dependent Kondner model, especially for the cases of very low strain rates which relate to the polymer pressure pipe applications.

## 1. Introduction

PE100 is the third generation of pipe grade high-density polyethylene (HDPE) and has an optimum balance of long-term strength, creep resistance, and slow crack growth resistance. PE100 pipes have been widely used in water and gas supply networks [1,2,3]. It is well recognized that HDPE exhibits viscoelastic–plastic behavior at room temperature, and its stress–strain response is strongly affected by the loading rate [4,5,6]. The yield stress and strain hardening modulus are reported to increase linearly with increasing logarithmic value of the strain rate [7].

As the deformation rate is very low for PE100 pipes subjected to medium or low internal pressure, the long-term deformation behavior and failure time are difficult to measure by tests. Moreover, reliable prediction models for the strength and failure of pressure pipes are still lacking [1,8,9,10]. In engineering practice, the tensile properties of the pipe material are measured in accordance with the ISO 527 standard at various test speeds ranging from 0.125 mm/min to 500 mm/min, depending on the test specimen size and test material type, and used to check the failure of the pipe accordingly. Numerous studies have shown that the strength of polymers, including HDPE, decreases with decreasing strain rate and with increasing temperature [4,11,12]; for instance, El-Bagory et al. [13] demonstrated that the room-temperature yield stress of an HDPE standard specimen with a gage length of 50 mm is 27.24 MPa when the loading speed is set to be 500 mm/min, while it decreases to 19.58 MPa under a loading speed of 5 mm/min. Thus, it is not conservative to directly use the strength tested under the laboratory loading rate, which is much higher than the creep rate in the real pipe case under a constant internal pressure, to evaluate the long-term strength of the pipe material. To properly characterize the long-term mechanical properties and the long-term strength of the pipe material, there are usually two methodologies: one is to perform long-term tensile tests with very slow speed, which are so expensive and time-consuming that few laboratories carry out such tests; the other is to predict the stress–strain response at very low strain rates through the combination of short-term laboratory tests at various strain rates and reliable rate-dependent constitutive models. This study adopts the latter method.

In recent decades, numerous macromechanical and micromechanical constitutive models have been proposed for describing the rate-dependent viscoelastic and viscoplastic behaviors of polymer materials. In micromechanical models, the crystalline and amorphous phases in semicrystalline polymers, including HDPE, are separately modeled owing to their different specific characteristics [14], and the two phases are combined to form a homogenized model that represents the bulk material; therefore, the micromechanical models are capable of studying the link between the mechanical properties and the chemical composition of the material [15]. Macromechanical models are generally phenomenological models, and the differential or integral constitutive equations are expressed as functions of stress, strain rate, and strain or time [16]. The viscoelastic response is often described by the Schapery single integral nonlinear viscoelastic model [17] or generalized Kelvin model in the differential form [18,19], and the viscoplastic response is often described by the overstress theory [20,21]. The model parameters are determined from creep, stress relaxation, monotonic and cyclic uniaxial testing at different strain rates.

This study focuses on developing a simple rate-dependent model from which the tensile properties of HDPE material can be derived. To achieve this goal, quasi-static tensile tests under constant strain rates ranging from 10^−5^ s^−1^ to 5 × 10^−2^ s^−1^ were conducted on PE100 pipe material, and the corresponding rate-dependent stress–strain curves were described by a modified hyperbolic constitutive model in which the rate dependences of the yield stress and the initial elastic modulus were considered.

## 2. Material and Tests

### 2.1. Material and Specimen

**Figure 1:**
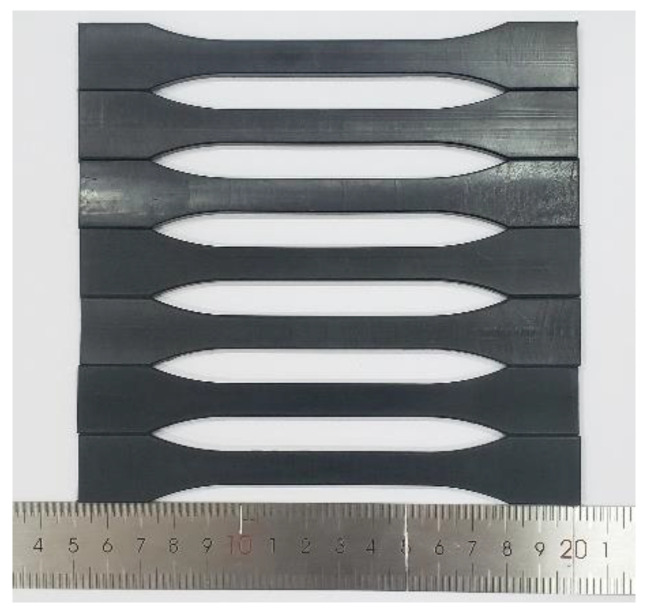
Tensile specimens of PE100 pipe material.

The specimens for tensile tests are cut and prepared from a PE100 gas pipe with dimensions of 315 mm in outer diameter and 28.6 mm in thickness, which is extrusion molded by Hebei Yisu Pipeline Co., Ltd. (Cangzhou, China) with the SABIC^®^ HDPE P6006 compound (SABIC Innovative Plastics (Shanghai) Co., Ltd., Shanghai, China) containing a carbon black content of 2.25%. The melt flow rate of the compound at 190 °C and 5 kg load is 0.23 g/10 min under the test specification of ISO 1133, and the density is measured to be 959 kg/m^3^ at 23 °C according to ASTM D1505. The shape and dimensions of the specimen shown in Figure 1 meet the requirements for the type B1 specimen recommended by ISO 527-2.

### 2.2. Tensile Tests with Constant Strain Rate

**Figure 2:**
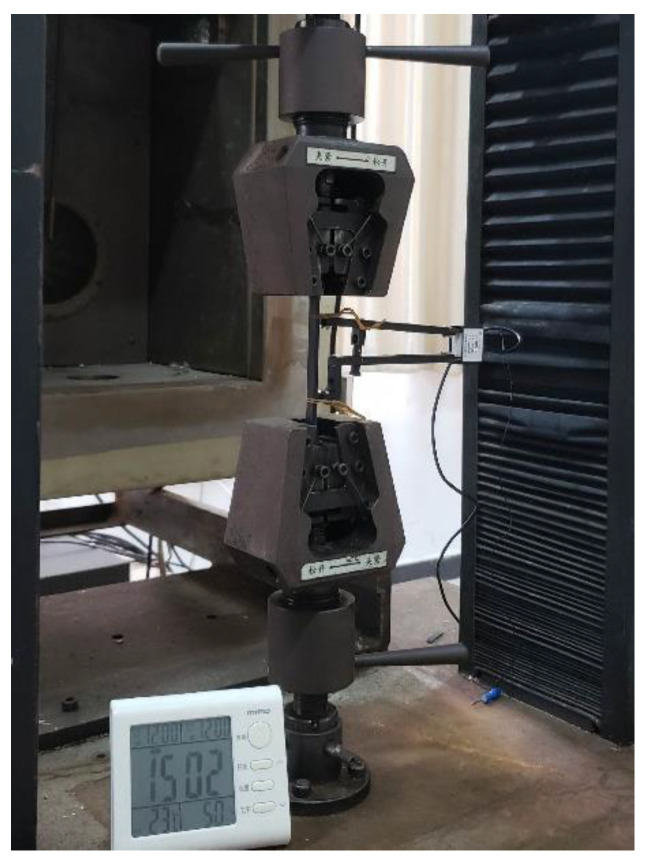
Tensile test setup.

**Figure 3:**
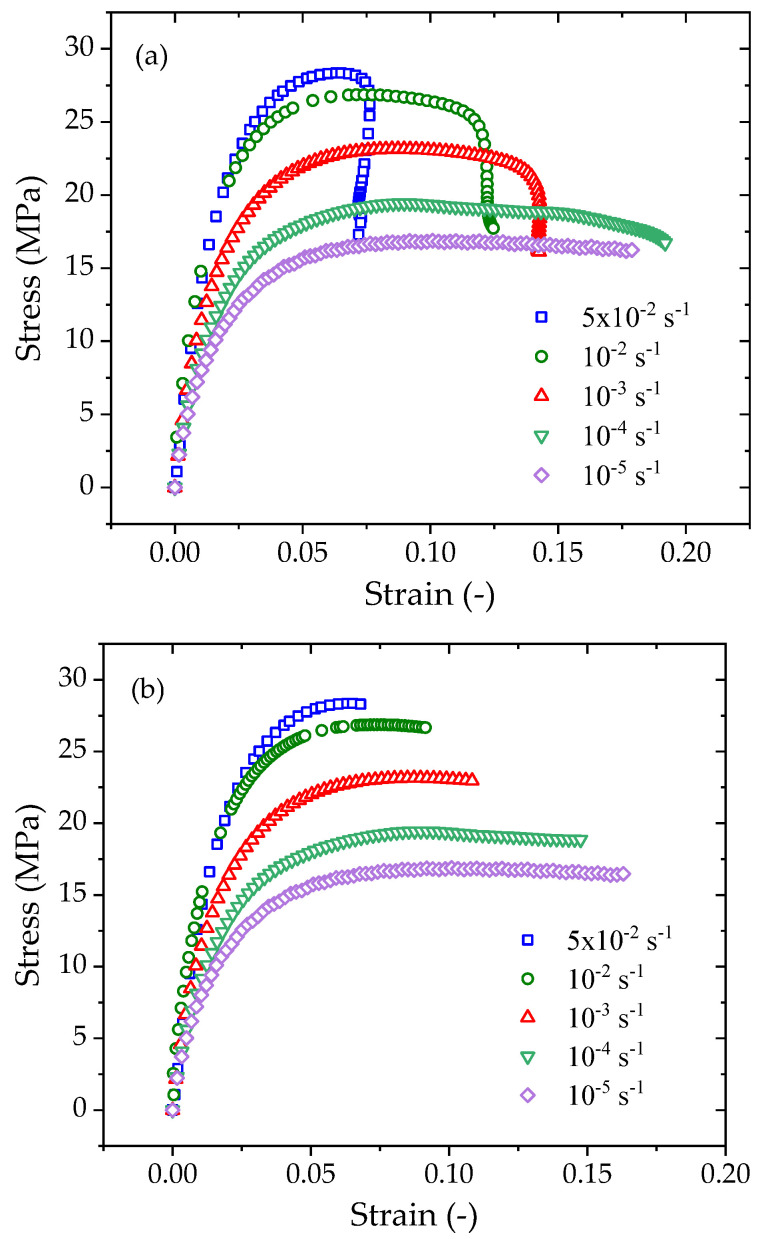
The representative engineering stress–strain curves of PE100 pipe material under various strain rates: (**a**) including yield necking behavior; (**b**) prior to yield necking.

**Figure 4:**
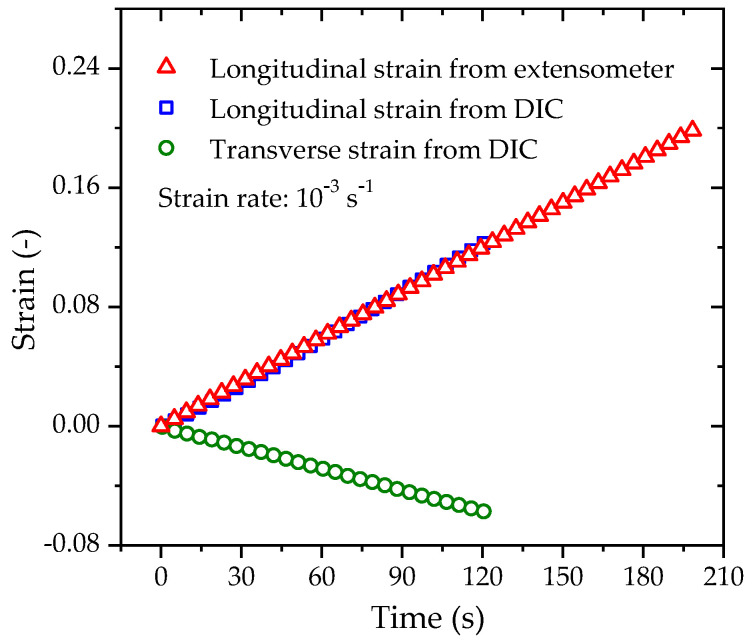
Variations of longitudinal and transverse strains with time for the PE100 pipe material loaded at a strain rate of 10^−3^ s^−1^.

The constant strain rate (CSR) tensile tests are completed on a CSS44020 electronic tensile testing machine, as shown in Figure 2. The specimens are stretched with five specified strain rates of 5 × 10^−2^ s^−1^, 10^−2^ s^−1^, 10^−3^ s^−1^, 10^−4^ s^−1,^ and 10^−5^ s^−1^; all tests are performed at 23 °C and at a relative humidity of 50% RH. The longitudinal strain is measured by an extensometer, and the digital image correlation (DIC) technique is simultaneously used to record the longitudinal and transverse deformations to evaluate the Poisson’s ratio during stretching. The CSR tests for each specified strain rate are repeated three times, and the average yield stress and yield strain are obtained for further model validations. The representative engineering stress–strain curves obtained from the tests are shown in Figure 3a. When the strain increases to a certain value, stress softening and strain localization occur due to necking. The subsequent stress–strain curve cannot truly reflect the constitutive characteristics of the material due to the nonuniform deformation. Therefore, we only retain the engineering stress–strain curve before necking for further analysis, as shown in Figure 3b. The longitudinal strain vs. time curve and the transverse strain vs. time curve measured under a strain rate of 10^−3^ s^−1^ are shown in Figure 4. The longitudinal strain measured by the extensometer is consistent with that measured by DIC technology. Therefore, in the other CSR tensile tests, only an extensometer is used to measure the longitudinal strain for the stress–strain response analysis.

## 3. Results and Discussions

### 3.1. Poisson’s Ratio

**Figure 5:**
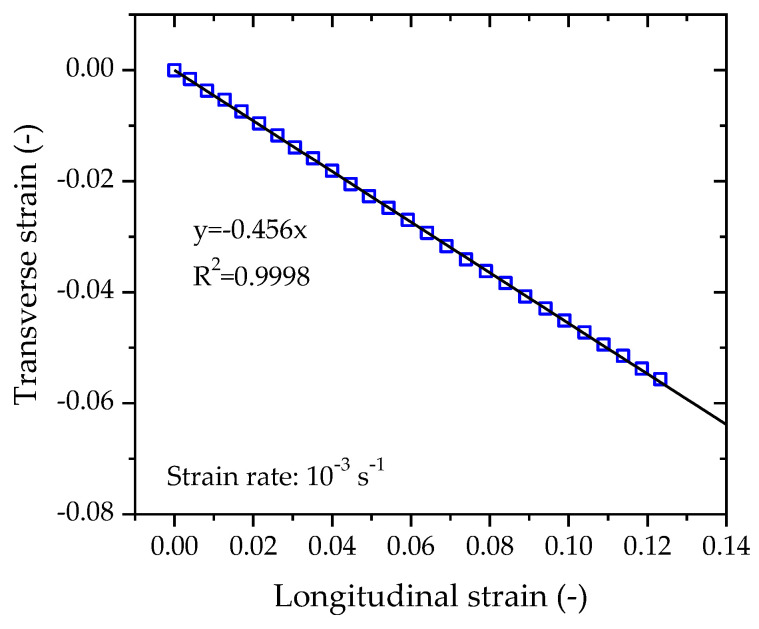
Transverse strain vs. longitudinal strain curve of PE100 pipe material loaded at a strain rate of 10^−3^ s^−1^.

Poisson’s material ratio can be measured by the negative ratio of transverse strain to longitudinal strain. The transverse strain and the longitudinal strain data set shown in Figure 4 are replotted in Figure 5. It is obvious that the transverse strain changes in proportion with the longitudinal strain during the tensile process. The data set is linearly fitted by a linear function revealing a straight line with a slope of −0.456; thus, the Poisson’s ratio of the tested PE100 pipe material is determined to be 0.456.

### 3.2. True Stress–Strain Curves

Figure 3 shows that the strains prior to necking yield reach 0.07–0.17 when loaded at strain rates from 10^−5^ s^−1^ to 5 × 10^−2^ s^−1^. As the specimens experience large deformation to yield, the corresponding true stress–strain curves are considered in the following analysis.

For the test data shown in Figure 3b, the engineering stress and strain are converted to the true stress and strain by Equations (1) and (2):

(1) $$\varepsilon_{\mathrm{true}}=\ln\left(1+\varepsilon \right)$$

(2) $$\sigma_{\mathrm{true}}=\sigma {\left(1+\varepsilon \right)}^{2\mu }$$

where $\varepsilon_{\mathrm{true}}$ and ε denote the true strain and the engineering strain, respectively; $\sigma_{\mathrm{true}}$ and σ denote the true stress and the engineering stress, respectively; and μ is the Poisson’s ratio of the material.

**Figure 6:**
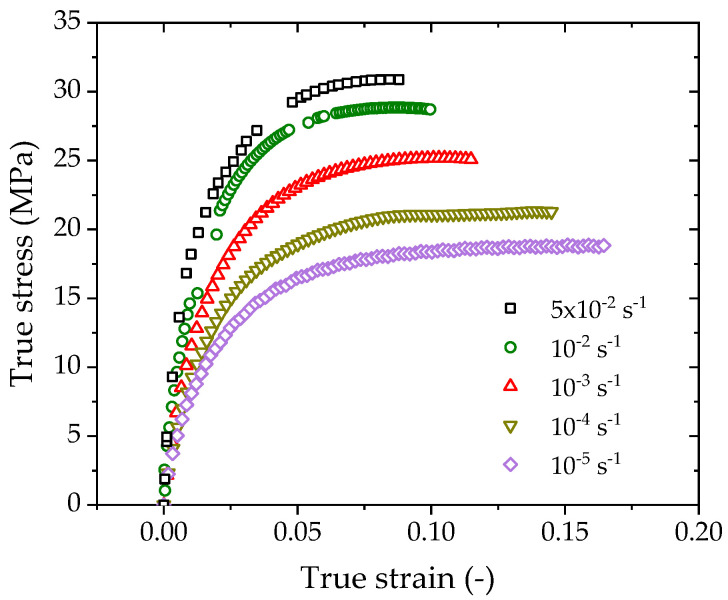
True stress—true strain curves of PE100 pipe material under various strain rates.

The converted true stress-true strain curves for various strain rates are plotted in Figure 6. The true stress at a given true strain increases with increasing strain rate. For instance, the true stresses at a true strain of 0.07 are 17.6 MPa, 20.4 MPa, 24.5 MPa, 28.6 MPa, and 30.6 MPa for strain rates of 10^−^^5^ s^−^^1^, 10^−^^4^ s^−^^1^, 10^−^^5^ s^−^^1^, 10^−^^6^ s^−^^1^, and 5 × 10^−^^5^ s^−^^1,^ respectively. For each order of magnitude increase in the strain rate, the true stress increases by 15–20%.

### 3.3. Rate-Dependent Yielding

**Figure 7:**
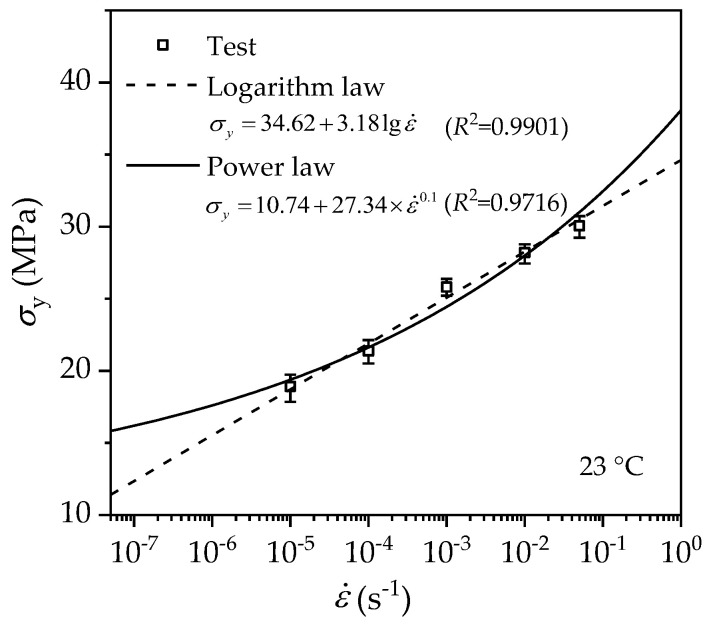
Variation of the true yield stress with a strain rate of PE100 pipe material.

As necking and strain softening are the consequence of yielding, the peak stress prior to necking is taken as the yield stress. The yield stresses at various strain rates can be determined from the test data in Figure 3, and Figure 7 shows the measured yield stress at five different strain rates. Two models are adopted to fit the data: one is the logarithm law, and the other is the power law.

#### 3.3.1. Logarithm Law

As suggested by the Eyring model [22,23], which was first proposed to describe the chemical reaction rate behavior and is currently applied to the viscoelasticity of polymers, the necking of polymers is considered as a thermal activation rate process; then, the yield stress and the logarithm of the strain rate satisfy a linear relationship as follows [24]:

(3) $$\sigma_{y}=\frac{k_{B}T}{V}\left[\ln\left(\frac{2\dot{\varepsilon }}{{\dot{\varepsilon }}_{0}}\right)+\frac{Q}{k_{B}T}\right]=A\mathrm{lg}\dot{\varepsilon }+B$$

where ${\dot{\varepsilon }}_{0}$ is the reference strain rate, $k_{B}$ is the Boltzmann constant, *T* denotes the temperature in Kelvin, Q denotes the activation energy, V is the activation volume, and *A* and *B* are material parameters. Fitting Equation (3) with the test data in Figure 7, the material parameters can be determined, as shown in Equation (4), with a correlation coefficient greater than 0.99. The dashed line in Figure 7 represents such regression fitting.

(4) $$\sigma_{y}=34.62+3.18\mathrm{lg}\dot{\varepsilon }$$

This logarithm-law rate dependence of the yield stress is consistent with a previous investigation by Zhang et al. [7].

#### 3.3.2. Power Law

Often, the rate dependence of the yield stress of polymers is instead described with a power law added to a constant [25]:

(5) $$\sigma_{y}=\sigma_{y0}+p{\dot{\varepsilon }}^{n}$$

where $\sigma_{y0}$ is the static yield stress corresponding to zero-rate tension (strain rate approaching zero), p and *n* are the coefficient and the exponent of the power-law dependence, respectively. The parameters are determined by curve fitting with the least-square-error method, as given in Equation (6). Such a fit provides a good description of the test data with a correlation coefficient greater than 0.97, as depicted by the solid line in Figure 7.

(6) $$\sigma_{y}=10.74+27.34{\dot{\varepsilon }}^{0.1}$$

## 4. Constitutive Model

### 4.1. Rate-Dependent Hyperbolic Model

The hyperbolic stress–strain model of Kondner [26] and Duncan and Chang [27], which was originally formulated for consolidated-undrained triaxial compression of soils, has been proven to be applicable for the nonlinear mechanical behavior of polymer materials [28,29]. The hyperbolic constitutive model is given as:

(7) $$\sigma_{1}-\sigma_{3}=\frac{\varepsilon }{a+b\varepsilon }$$

in which $\sigma_{1}$ and $\sigma_{3}$ are the major and minor principal stresses, ε is the axial strain, and a and b are experimentally determined material constants that are generally considered as uncorrelated independent variables. Suleiman et al. [29] further considered the linear correlation between the parameters a and b, and developed a focus point approach to estimate the parameters. The original Kondner hyperbolic model does not consider the strain rate effect of the material in the constitutive relationship.

To consider the strain-rate dependent behavior, the material parameters a and b are assumed to be strain-rate dependent due to the viscoelasticity of the material [28]. Thus, for uniaxial tension of polymer materials, Equation (7) reduces to

(8) $$\sigma \left(\varepsilon ,\dot{\varepsilon }\right)=\frac{\varepsilon }{a\left(\dot{\varepsilon }\right)+b\left(\dot{\varepsilon }\right)\cdot \varepsilon }$$

The mechanical meanings of the two parameters are considered below. If the strain approaches infinity, the stress will reach its asymptotic value, which is called the ultimate stress $\sigma_{u}$, i.e., $\sigma_{u}\left(\dot{\varepsilon }\right)=\underset{\varepsilon \to \infty }{\lim}\sigma =1/b\left(\dot{\varepsilon }\right)$. It is clear that the ultimate stress derived from Equation (8) is higher than the maximum stress that the real material experienced during tension as no real material can deform to infinite strain before fracture. For the PE100 pipe material in this study, the maximum stress is the yield stress prior to necking; thus, the yield stress is assumed to relate to the ultimate stress in the following relation:

(9) $$\sigma_{y}\left(\dot{\varepsilon }\right)=r\cdot \sigma_{u}\left(\dot{\varepsilon }\right),\;\left(r<1\right)$$

where *r* is the ratio of the yield stress to the fictitious ultimate stress. Therefore, parameter *b* is proportional to the reciprocal of the yield stress of the material.

The strain-dependent tangent modulus is obtained through the derivative of Equation (8) with respect to strain:

(10) $$E_{t}=\frac{\partial \sigma }{\partial \varepsilon }=\frac{a\left(\dot{\varepsilon }\right)}{{\left[a\left(\dot{\varepsilon }\right)+b\left(\dot{\varepsilon }\right)\cdot \varepsilon \right]}^{2}}$$

where $E_{t}$ is the tangent modulus. The initial modulus is defined as

(11) $$E_{0}\left(\dot{\varepsilon }\right)=\underset{\varepsilon \to 0}{\lim}E_{t}=\frac{1}{a\left(\dot{\varepsilon }\right)}$$

Because of the large deformation mentioned in Section 3.2, the true stress and true strain are used to describe the mechanical behavior of the PE100 pipe material. Replacing the stress *σ* and the strain *ε* in Equation (8) with the corresponding true stress $\sigma_{\mathrm{true}}$ and true strain $\varepsilon_{\mathrm{true}}$, the hyperbolic constitutive model with two rate-dependent parameters, $a\left(\dot{\varepsilon }\right)$ and $b\left(\dot{\varepsilon }\right)$ or $E_{0}\left(\dot{\varepsilon }\right)$ and $\sigma_{y}\left(\dot{\varepsilon }\right)$, is rewritten as

(12) $$\sigma_{\mathrm{true}}=\frac{\varepsilon_{\mathrm{true}}}{a\left(\dot{\varepsilon }\right)+b\left(\dot{\varepsilon }\right)\cdot \varepsilon_{\mathrm{true}}}=\frac{\varepsilon_{\mathrm{true}}}{\frac{1}{E_{0}\left(\dot{\varepsilon }\right)}+\frac{r}{\sigma_{y}\left(\dot{\varepsilon }\right)}\varepsilon_{\mathrm{true}}}$$

In engineering applications, yielding is often considered one of the failure criteria. In such cases, the failure strain corresponding to the yield stress is given by

(13) $$\varepsilon_{y}\left(\dot{\varepsilon }\right)=\frac{\sigma_{y}\left(\dot{\varepsilon }\right)}{\left(1-r\right)E_{0}\left(\dot{\varepsilon }\right)}$$

### 4.2. Identification of Model Parameters

Equation (12) can be rewritten in a linearized form as follows:

(14) $$\frac{\varepsilon_{\mathrm{true}}}{\sigma_{\mathrm{true}}}=a\left(\dot{\varepsilon }\right)+b\left(\dot{\varepsilon }\right)\cdot \varepsilon_{\mathrm{true}}=\frac{1}{E_{0}\left(\dot{\varepsilon }\right)}+\frac{r}{\sigma_{y}\left(\dot{\varepsilon }\right)}\varepsilon_{\mathrm{true}}$$

**Figure 8:**
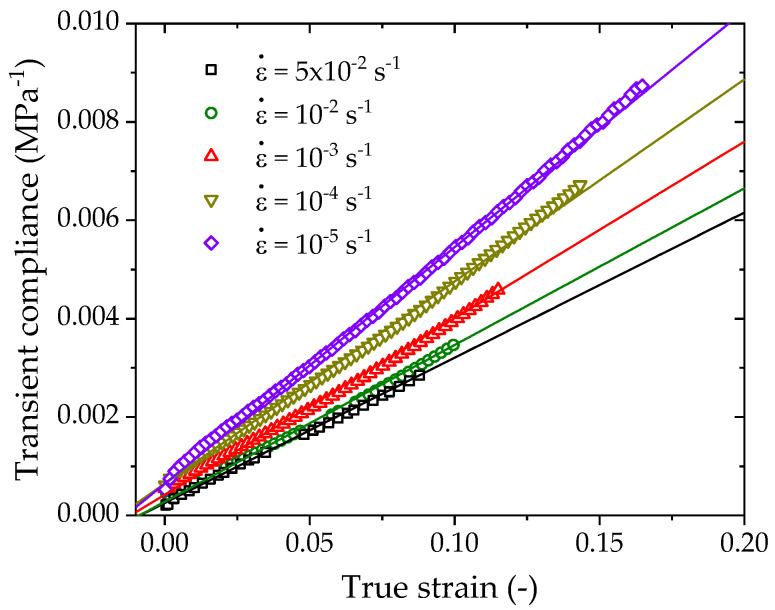
Transient compliance-true strain curves of PE100 pipe material under various strain rates.

In this transformation, the ratio of the true strain to the true stress defines the transient compliance, a or $1/E_{0}$ represents the intercept and *b* or $r/\sigma_{y}$ represents the slope of the transient compliance versus true strain curve. Figure 8 shows the transient compliance—true strain curves of PE100 pipe material under various strain rates by transforming from the data in Figure 6. The linearity of this transformation is confirmed by the linear regression lines for each strain rate, which are highly consistent with the experimental data.

Substituting Equation (4) into Equation (14), the linear regression lines determine the parameters $E_{0}\left(\dot{\varepsilon }\right)$, $a\left(\dot{\varepsilon }\right)$ and *r*, which are listed in Table 1. All regressions have high correlation coefficients that are not less than 0.99. The ratio *r* has a rate independent value of 0.9.

**Figure 9:**
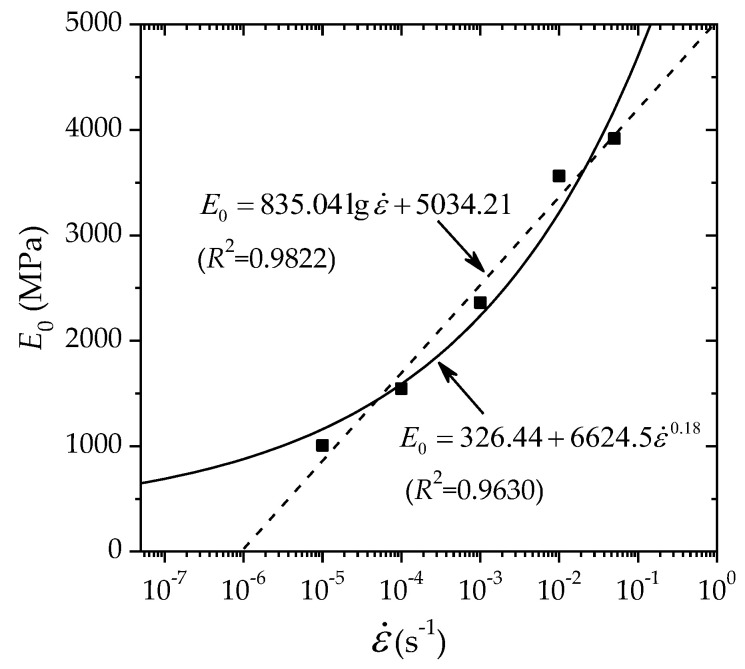
Variation of the initial elastic modulus with the strain rate.

It is also necessary to obtain the relationship between the initial modulus and the strain rate. Figure 9 shows the variation of the initial modulus with the strain rate. Two types of functions are used for regression fitting: the power law and the logarithm law. The regressions are given in Equations (15) and (16). It is seen that two regressions have high correlation coefficients greater than 0.96; however, Equation (16) is not appropriate for the cases of extremely low strain rates, as it predicts a negative modulus for strain rates less than 10^−6^ s^−1^. Equation (15) reveals that the initial elastic modulus increases with increasing strain rate by the power-law with a constant static value.

(15) $$E_{0}\left(\dot{\varepsilon }\right)=326.44+6624.5{\dot{\varepsilon }}^{0.18}\begin{array}{cc} & \left(R^{2}=0.9630\right) \end{array}$$

(16) $$E_{0}\left(\dot{\varepsilon }\right)=5034.21+835.04\mathrm{lg}\dot{\varepsilon }\begin{array}{ccc} & & \left(R^{2}=0.9822\right) \end{array}$$

### 4.3. Determined Rate-Dependent Constitutive Model

Two optional rate-dependent hyperbolic constitutive models for PE100 pipe material are finally determined by selecting either the logarithm-law or the power-law dependence.

#### 4.3.1. Model with Logarithm-Law Rate Dependence

Substituting Equations (4) and (16) into Equation (12) and setting *r* = 0.9, the logarithm-law rate-dependent hyperbolic constitutive model is finally determined by

(17) $$\sigma_{\mathrm{true}}\left(\dot{\varepsilon }\right)=\frac{\varepsilon_{\mathrm{true}}}{\frac{1}{5034.21+835.04\mathrm{lg}\dot{\varepsilon }}+\frac{0.9}{34.62+3.18\mathrm{lg}\dot{\varepsilon }}\varepsilon_{\mathrm{true}}}$$

**Figure 10:**
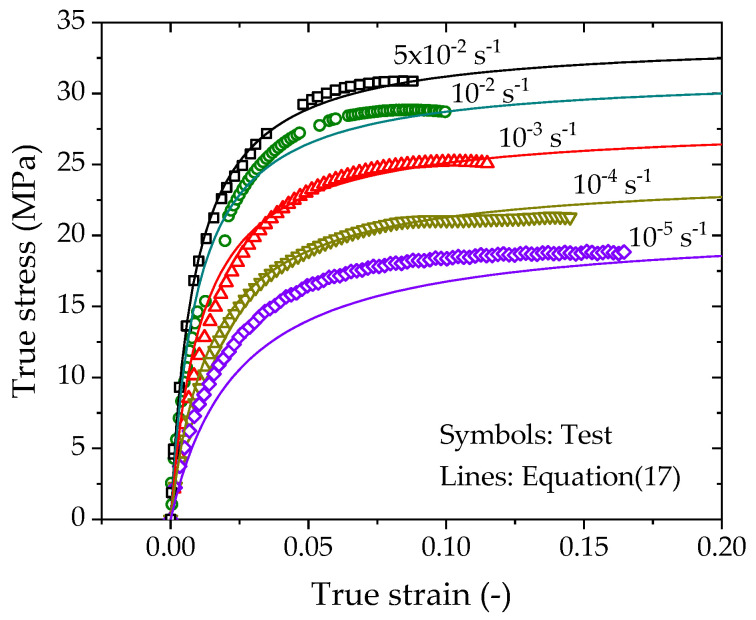
True stress vs. true strain curves predicted by the logarithm-law rate-dependent model.

The predictions by Equation (17) are plotted as solid lines in Figure 10, which are in good agreement with the experimental data in the CSR tests except for the case of 10^−5^ s^−1^. The inconsistency is attributed to the underestimation of $E_{0}$ and $\sigma_{y}$ for low strain rates less than 10^−5^ s^−1^.

#### 4.3.2. Model with Power-Law Rate Dependence

Substituting Equations (6) and (15) into Equation (12) and setting *r* = 0.9, the power-law rate-dependent hyperbolic constitutive model is finally determined by

(18) $$\sigma_{\mathrm{true}}\left(\dot{\varepsilon }\right)=\frac{\varepsilon_{\mathrm{true}}}{\frac{1}{326.44+6624.5{\dot{\varepsilon }}^{0.18}}+\frac{0.9}{10.74+27.34{\dot{\varepsilon }}^{0.1}}\varepsilon_{\mathrm{true}}}$$

**Figure 11:**
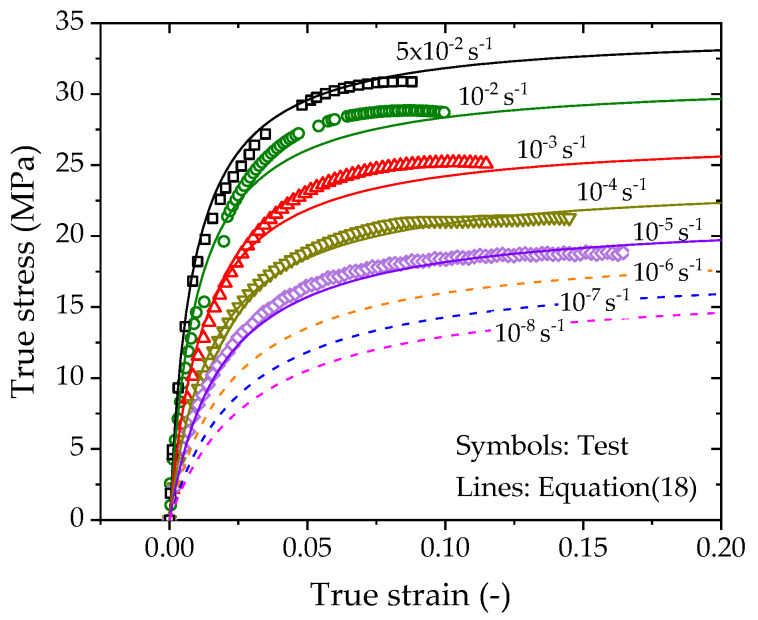
True stress vs. true strain curves predicted by the logarithm-law rate-dependent model.

The predictions by Equation (18) are plotted as solid and dashed lines in Figure 11. The solid lines are consistent with the experimental data in the CSR tests under all strain rates, while the dashed lines show the reasonable predictions of stress–strain responses for the case of very low strain rates.

Furthermore, the predictions for yield strains under various strain rates are given by substituting either Equations (4) and (16) or Equations (6) and (15) into Equation (13) and setting *r* = 0.9.

Logarithm-law rate-dependent yield strain:

(19) $$\varepsilon_{y}\left(\dot{\varepsilon }\right)=\frac{34.62+3.18\mathrm{lg}\dot{\varepsilon }}{\left(1-0.9\right)\times \left(5034.21+835.04\mathrm{lg}\dot{\varepsilon }\right)}$$

Power-law rate-dependent yield strain:

(20) $$\varepsilon_{y}\left(\dot{\varepsilon }\right)=\frac{10.74+27.34{\dot{\varepsilon }}^{0.1}}{\left(1-0.9\right)\times \left(326.44+6624.5{\dot{\varepsilon }}^{0.18}\right)}$$

**Figure 12:**
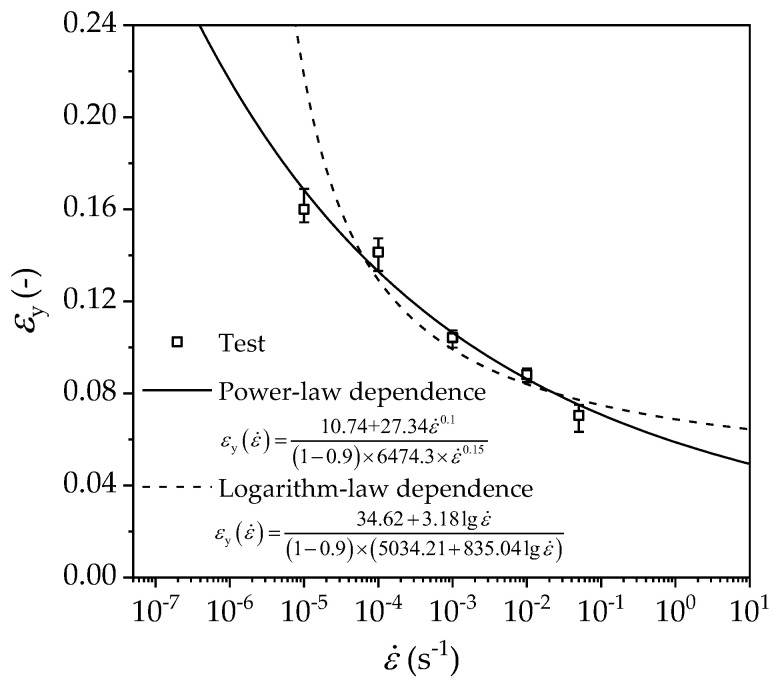
Comparison of predicted and measured yield strain vs. strain rate relationship.

As shown in Figure 12, the predictions for yield strain by Equations (19) and (20) generally agree with the experimental measurements. In contrast, Equation (20) is more appropriate than Equation (19).

## 5. Conclusions

The investigation performed uniaxial tension tests at five specified strain rates ranging from 10^−5^ s^−1^ to 5 × 10^−2^ s^−1^ on PE100 pipe material. The strain rate dependence of the yield stress and initial tangent modulus were analyzed and incorporated into the rate-dependent Kondner hyperbolic constitutive model. The following conclusions can be drawn from the present investigation:

(a) The tensile mechanical behavior of PE100 pipe material depends on the loading strain rate, and the strain-rate dependence of the yield stress and the initial tangent modulus can be described by either a power law or a logarithm law in the tested strain rate range.
(b) The strain-rate dependent Kondner hyperbolic constitutive model takes the yield stress and initial tangent modulus of the material as model parameters, and it can describe the tensile mechanical behavior of PE100 pipes prior to yielding under various strain rates. The predictions agree well with the tests and provide the stress–strain responses at very low strain rates. In contrast, the power-law rate-dependent Kondner model is more suitable for describing the rate-dependent tensile behavior of PE100 pipe than the logarithm-law rate-dependent Kondner model.

## Figures and Tables

**Table 1:** Model parameters *E*_0_, *a* and *r*.

$\dot{\varepsilon }$/s^−1^	*E*_0_/MPa	*a*/MPa^−1^	*r*	Correlation Coefficient, *R*^2^
10^−5^	1.26 × 10^3^	7.93 × 10^−^^4^	0.90	0.9991
10^−4^	1.55 × 10^3^	6.47 × 10^−^^4^	0.90	0.9981
10^−3^	2.36 × 10^3^	4.23 × 10^−^^4^	0.90	0.9974
10^−2^	3.56 × 10^3^	2.81 × 10^−^^4^	0.90	0.9977
5 × 10^−2^	3.92 × 10^3^	2.55 × 10^−^^4^	0.90	0.9995

## Data Availability

The data presented in this study are available on request from the corresponding author.
